# Effectiveness of ultra-long-term lithium treatment: relevant factors and case series

**DOI:** 10.1186/s40345-024-00328-9

**Published:** 2024-03-15

**Authors:** Ewa Ferensztajn-Rochowiak, Ute Lewitzka, Maria Chłopocka-Woźniak, Janusz K. Rybakowski

**Affiliations:** 1 Department of Adult Psychiatry, Poznan University of Medical Sciences, Poznan, Poland; 2 Department of Psychiatry and Psychotherapy, Universitätsklinikum Carl Gustav Carus, Dresden, Germany

**Keywords:** Lithium, Bipolar disorder, Ultra-long-term prophylaxis

## Abstract

**Background:**

The phenomenon of preventing the recurrences of mood disorders by the long-term lithium administration was discovered sixty years ago. Such a property of lithium has been unequivocally confirmed in subsequent years, and the procedure makes nowadays the gold standard for the pharmacological prophylaxis of bipolar disorder (BD). The efficacy of lithium prophylaxis surpasses other mood stabilizers, and the drug has the longest record as far as the duration of its administration is concerned. The continuation of lithium administration in case of good response could be a lifetime and last for several decades. The stability of lithium prophylactic efficacy in most patients is pretty steady. However, resuming lithium after its discontinuation may, in some patients, be less efficient.

**Main body:**

In the article, the clinical and biological factors connected with the prophylactic efficacy of long-term lithium administration are listed. Next, the adverse and beneficial side effects of such longitudinal treatment are presented. The main problems of long-term lithium therapy, which could make an obstacle to lithium continuation, are connected with lithium’s adverse effects on the kidney and, to lesser extent, on thyroid and parathyroid functions. In the paper, the management of these adversities is proposed. Finally, the case reports of three patients who have completed 50 years of lithium therapy are described.

**Conclusions:**

The authors of the paper reckon that in the case of good response, lithium can be given indefinitely. Given the appropriate candidates for such therapy and successful management of the adverse effects, ultra-long term lithium therapy is possible and beneficial for such patients.

## Historical background

In 2023, we observe sixty’s anniversary of the publication, where, for the first time in modern psychiatry, the possibility of preventing recurrences of mood disorders by the long-term application of lithium salts was described. The paper’s author was Geoffrey Philip Hartigan (1917–1968), known as „Toby”, a British psychiatrist at St. Augustine’s Hospital in Chartham Down. Among forty-five patients treated with lithium carbonate in the hospital within the last six years, he summarized his observations on long-term lithium administration (≥ 3 years) in seven patients with bipolar mood disorder (BD) and eight with recurrent depression. In six persons from the first group (86%) and six from the second group (75%), there were no recurrences of the illness during this period (Hartigan 1963). This publication appeared fourteen years after the article of an Australian psychiatrist, John Cade, showing the therapeutic effect of lithium in manic states and regarded as a harbinger of introducing lithium into contemporary psychiatry (Cade 1949). In 1964, a Danish psychiatrist, Poul Christian Baastrup (1918–2002), depicted observations on the „prophylactic” effect in eleven BD patients taking lithium for three years (Baastrup 1964).

Although Hartigan’s paper can be considered seminal for showing the prophylactic efficacy of long-term lithium administration, the historical data from the nineteenth century provided the evidence that a Danish scientist, Carl Lange (1834–1900) was giving longitudinally lithium carbonate to patients with periodic depression. The rationale for using lithium was a concept of depression as “uric acid diathesis” (gout of the brain) (Lange, 1886). Previously, lithium was introduced to treat gout by a British physician, Alfred Garrod (1819–2007) (Garrod 1859). According to Felber (1987), during twenty years of practice, Lange could provide lithium to about a thousand patients, observing both therapeutic and prophylactic effects in those with periodic depression.

The pioneering idea of Hartigan was to indicate the possibility of a favorable effect of lithium administration on the long-term course of mood disorders, both bipolar and unipolar. He referred to the names „normothymotics” and „mood-normalizers”, which were proposed by a Danish psychiatrist, Mogens Schou (1918–2005), for lithium and imipramine, as the drugs normalizing mood in BD and periodic depression, respectively (Schou 1963). The preventive action lithium against both manic and depressive recurrences revealed by Hartigan justified naming it a drug which normalizes (stabilizes) the mood. Therefore, lithium became a precursor of drugs defined as “mood stabilizers”, and such a name has presently been used worldwide. However, in Polish and Russian psychiatric literature, the term „normothymic drugs” has also been employed.

In 1967, a paper appeared by Danish psychiatrists mentioned before (Baastrup and Schou) summing up the lithium administration for a mean of 6 years to eighty-eight patients with bipolar and unipolar mood disorders treated in Glostrup Psychiatric Hospital. The results showed that the mean duration of abnormal mood (mania or depression) during lithium treatment was more than six-fold shorter compared to the period before lithium, indicating a prophylactic effect of lithium on the course of mood disorders with a high probability (Baastrup and Schou 1967)_._ In 1968, a highly critical article against the possibility of lithium prophylaxis appeared, published in the prestigious journal „Lancet”, authored by the British psychiatrists Barry Blackwell and Michael Shepherd and titled: „Prophylactic lithium: another therapeutic myth?”. The authors were intensely dubious about the results of Danes and postulated performing double-blind studies to verify the effect of lithium (Blackwell and Shepherd 1968).

In reply to the recommendation of the Britons, in 1970–1973, eight placebo-controlled studies on the prophylactic efficacy of lithium were performed in Europe (Denmark and the UK) and the USA, with patients who had in two years of pre-lithium period at least two episodes of the illness. The researchers mostly made comparisons between subjects in which lithium was replaced by a placebo and those continuing lithium (so-called “discontinuation design”). The overall analysis revealed that the percentage of patients with recurrences of depression or mania was significantly lower during lithium administration (mean 30%) than during placebo (mean 70%) (Schou and Thomsen, 1975). These results paved the way for the widespread application of lithium for prophylactic purposes, predominantly in BD, during the 1980s and 1990s. In the twenty-first century, the recurrence-preventing properties of lithium in BD were confirmed again in the meta-analyses. They corroborated a robust prophylactic effect of lithium against manic and moderate against depressive recurrences (Geddes et al. 2004; Nivoli et al. 2010; Severus et al. 2014).

The studies also compared the prophylactic effect of lithium in BD and that of other mood-stabilizing drugs of the first generation (carbamazepine, valproate), and the second generation (lamotrigine, olanzapine, quetiapine) (Rybakowski 2023). The MAP project (Multicenter study of long-term treatment of Affective or schizoaffective Psychoses) comparing lithium and carbamazepine for two-and-half years showed the advantage of lithium in classic BD and that of carbamazepine in atypical form of the illness (Kleindienst and Greil 2000). The BALANCE study (Bipolar Affective disorder Lithium/ANtiConvulsant Evaluation) lasting two years demonstrated a better prophylactic effect of lithium than valproate and the best efficacy combining of both drugs (Geddes et al. 2010). A one-and-half-year comparison of lithium and lamotrigine showed better prevention by lithium of manic episodes while depressive ones by lamotrigine (Goodwin et al. 2004). Somewhat opposite results were obtained with the one-year comparison of lithium with olanzapine, where the latter exerted a better effect against mania, while lithium—against depression (Tohen et al. 2005). A 4 year observation showed significantly better prophylactic effect of lithium than quetiapine (Altamura. 2008). An analysis by Kessing et al. (2018) revealed that in BD population, lithium monotherapy is prophylactically more effective compared with monotherapy of other mood-stabilizing drugs such as valproate, lamotrigine, olanzapine, and quetiapine. Finnish investigators’ recent survey of real-world effectiveness of pharmacological treatments for BD patients showed that lithium was the best drug for decreasing hospitalizations for both psychiatric and somatic reasons (Lähteenvuo et al. 2023).

Nowadays, lithium is regarded as the first-line drug for the long-term prophylaxis of recurrences in mood disorders (Fountoulakis et al. 2022). For such procedures, lithium has been indicated by most guidelines (e.g., Canadian Network for Mood and Anxiety Treatments and International Society of Bipolar Disorder (Yatham et al. 2018), and recommendations (Malhi et al. 2017). Also, lithium, being the first mood-stabilizing drug, has a record of the most prolonged administration for prophylactic purposes among all mood stabilizers, reaching up to several decades.

It is then legitimate to ask: How long should lithium be given? There is a concern for a possible decreased efficacy during long-term administration. Such tolerance was suggested in 9% of 129 patients, developing after an average of ten years in complete responders and fifteen years in partial responders (Koukopoulos et al. 1995). However, Berghӧfer et al, (2013), estimating the stability of long-term lithium treatment in 346 patients receiving the drug for 1–20 years, showed that in patients continuing the therapy, the morbidity index remained stable over time and was not associated with the duration of lithium treatment, the number and frequency of episodes previous to treatment and the latency from the onset of bipolar disorder to the start of lithium treatment. On the other hand, some patients receiving long-term lithium treatment choose to discontinue the drug for various reasons and, after some time, decide to resume it. It transpired that in a part of such patients (about 1/3), there is a decreased responsiveness to lithium during further administration (Post 2012; Cakir et al. 2017).

The authors of the paper reckon that in a case of good response, lithium should be given indefinitely. Here, we discuss the factors connected with prophylactic lithium efficacy as well as the adverse and beneficial side effects of its long-term treatment. We also present three cases of patients with half-century treatment with lithium.

## Clinical and biological factors connected with lithium efficacy

In 1999, a half-century following lithium introduction into contemporary psychiatry (Cade 1949), a Canadian psychiatrist of Czechoslovakian origin, Paul Grof, introduced a concept of „excellent lithium responders” (ELR) as the BD patients, in whom lithium monotherapy results in a total elimination of the illness' recurrences (Grof 1999). Two years later, the assessment of the percentage of ELR was performed in Poznań, showing that about one-third of BD patients were free of recurrences during ten years of lithium administration (Rybakowski et al. 2001). In his more recent paper, Grof (2010) speculates that ELR may have features similar to those meeting Kraepelinian concept of "manisch-depressives Irresein", such as periodicity with distinct intervals of remission and a lack of psychiatric and somatic co-morbidity.

The factors that could increase the probability of a favorable prophylactic lithium response have been the subjects of many research. The most frequent clinical features that transpired to correlate with good prophylactic effectiveness of lithium were an episodic course with periods of complete remission, pattern of mania-depression interval, absence of psychotic episodes, non-rapid cycling, later onset of illness, short duration of disease before lithium administration, and low psychiatric comorbidity (Kleindienst et al. 2005a; Hui et al. 2019; Nunes et al. 2020). Whereas, the study of Berghöfer et al. (2008), including 242 patients receiving lithium for 1–10 years, showed that the long-term response to lithium maintenance is as good in patients with typical as in those with atypical traits such as psychiatric co-morbidity, rapid cycling or mood incongruent psychotic symptoms.

In Poznan, the personality traits assessed by the Temperament Scale of Memphis, Pisa, Paris, and San Diego Auto questionnaire (TEMPS-A) and the Oxford-Liverpool Inventory of Feelings and Experiences (O-LIFE) were related to lithium’s prophylactic efficacy. Utilizing the TEMPS-A, a positive correlation with hyperthymic temperament and a negative one with anxious, cyclothymic, and irritable temperaments were observed (Rybakowski et al. 2013). Using the O-LIFE, a negative correlation was obtained with cognitive disorganization, which can bear a predilection to psychosis (Dembinska-Krajewska et al. 2012.

Among the social and demographic factors related to good outcome were high social status, social support, good compliance, and a psychological tendency to dominate (Kleindienst et al. 2005b). A history of at least two childhood traumatic events, such as emotional, physical, and sexual abuse, significantly increases the risk of poor response to lithium (Etain et al. 2017). The chance is also higher with the occurrence of post-traumatic stress disorder (Cakir et al. 2016).

As the clinical and psychological factors connected with lithium prophylactic efficacy are ready to be considered in clinical practice, applying biological ones may take some time. However, in recent years, there has been a lot of research in this field. For instance, studies on induced pluripotent stem cells (iPSCs) aimed to find the differences between such cells obtained from lithium responders vs. non-responders. Mertens et al. (2015) investigated the cellular phenotypes of hippocampal dentate gyrus-like neurons derived from iPCSs of patients with BD. The hyperexcitability phenotype of young neurons was selectively reversed by lithium treatment only in neurons derived from lithium-responding patients. Similarly, Stern et al. (2018), using iPSCs derived from Epstein-Barr virus immortalized B-lymphocytes, showed that chronic lithium treatment reduced the hyperexcitability of these cells in lithium responders but not in lithium non-responders. At the same time, Tobe et al. (2017) showed that lithium alters the phosphorylation state of collapsin response mediator protein-2 (CRMP2), and the ratio of phosphorylated CRMP2 (pCRMP2) to CRMP2 was significantly higher in lithium responders than in non-responders.

The quality of prophylactic lithium response has also become an essential area for molecular-genetic research. Up to the second decade of the twenty-first century, such studies have been dominated by the “candidate gene” strategy. In 2013, the candidate genes connected with the good prophylactic effect of lithium were reviewed (Rybakowski 2013). In the recent decade, many other candidate genes have been identified, including genes involved in stress response (Szczepankiewicz et al. 2018), and so-called “clock” genes (Rybakowski et al. 2014; Geoffroy et al. 2016). Concerning the latter, the study of McCarthy et al. (2019), which showed that lithium responders have a higher level of morning chronotype than lithium non-responders could be mentioned. This finding could relate to our study, where we found that lithium treatment brings a tendency to a morning chronotype (Dopierala et al. 2017).

In 2009, the National Institutes of Mental Health and the International Group for the Study of Lithium-treated Patients (IGSLI) created the International Consortium on Lithium Genetics (ConLiGen), aiming for the first genome-wide association study (GWAS) of lithium response (Schulze et al. 2010). The phenotypic measure used by ConLiGen has been the "Retrospective Criteria of Long-Term Treatment Response in Research Subjects with Bipolar Disorder" scale, known as Alda’s scale (Manchia et al. 2013, Scott et al. 2020). In the first GWAS of the prophylactic lithium efficacy, 2500 subjects participated, representing twenty-two centers. The criteria for association with lithium response were met by a single locus of four linked single nucleotide polymorphisms (SNPs) located on chromosome 21. This locus has two genes for long, non-coding RNAs (lncRNAs) managing central nervous system gene expression (Hou et al. 2016). When studying the contribution of micro-RNA (miR) to lithium prophylactic efficacy, the ConLiGen group showed an association of the miR-630 with continuous phenotype and the miR-607 with dichotomous one of Alda’s scale (Reinbold et al. 2018).

Many subsequent papers from the ConLiGen group estimated the association of lithium prophylactic efficacy with the polygenic risk score (PRS) for various psychiatric conditions. It was found that the PRS for schizophrenia determines the worse response to lithium (International Consortium on Lithium Genetics (ConLiGen) et al. 2018), which can correspond with our study on the negative association of prophylactic lithium effect with a predisposition to psychotic symptoms (Dembinska-Krajewska et al. 2012). Amare et al. (2021) showed that the PRS for major depression is also negatively correlated with lithium response. In the subsequent paper, it was shown that combining schizophrenia and depression PRSs may additionally improve the genetic prediction of lithium response (Schubert et al. 2021). A further upgrade can be obtained by combining the PRS for schizophrenia and depression with some clinical factors (Cearns et al. 2022). In the study of Coombes et al., (2021), the PRS score for attention-deficit/hyperactivity disorder (ADHD) was connected with a worse response to lithium, while the PRS for schizophrenia was associated with higher rates of nonadherence. The ConLiGen developed a polygenic score for lithium response and found its association with the Alda scale’s categorical and continuous patterns. Furthermore, within this research, 36 candidate genes connected mostly with glutamatergic and cholinergic systems were revealed (Amare et al. 2023). The ConLiGen group also studied an association of lithium response with Human Leukocyte Antigen (HLA) variants, showing that good response to lithium was associated with HLA-mediated low inflammation. In contrast, poor response was connected with an inflammatory status (Le Clerc et al. 2021).

The research of French investigators showed that biomarkers of response to lithium may be also identified through peripheral epigenetic measures. By using a genome-wide methylomic approach (SeqCapEpi) and finding differentially methylated regions (DMRs), it was possible to discriminate good responders from non-responders to lithium (Marie-Claire et al. 2020). The combination of three clinical features: polarity at onset, psychotic symptoms at onset, and family history of bipolar disorder classified correctly 70% of individuals according to their lithium response, but when combined with the epigenetic biomarkers, plus alcohol misuse, the model correctly typed 86% of individuals (Marie-Claire et al. 2022, 2023).

Much other biological and genetic research has been performed in recent decade aiming to find neurobiological differences between lithium responders and non-responders, as well as the new genes that could be associated with lithium’s prophylactic effect. Some results were obtained from studying the lymphoblastoid cell lines (Breen et al. 2016; Papadima et al. 2018; Milanesi et al. 2019), from the GWAS studies performed outside the ConLiGen group (Higgins et al. 2015; Song et al. 2016), and from the investigations employing diverse molecular-genetic methods (Stacey et al. 2018; Jacobs et al. 2020).

In Table 1, most clinical and biological factors of good response to lithium were listed.

**Table 1:** Clinical and biological factors of good lithium response

Clinical	Biological
Episodic course with periods of complete remissions Pattern of mania-depression-interval Absence of psychotic episodes Non-rapid-cycling course Later onset of illness Short duration of the illness before lithium administration Low psychiatric comorbidity Hyperthymic temperament High social status Social support Good compliance No history of childhood abuse	Different effect on induced pluripotent stem cells Different effect on lymphoblastoid cell lines Candidate genes, including genes involved in stress response and “clock” genes Genes for lncRNA on chromosome 21 (ConLiGen GWAS research) Worse response with polygenic risk score for schizophrenia, major depression and attention-deficit/hyperactivity disorder Genes for HLA-mediated low inflammation Differences in methylated regions (genome wide methylomic study)

## Adverse somatic effects of lithium administration

The administration of lithium may be connected with a number of adverse bodily effects. In the first period of lithium use, the most frequent are hand tremors, polyuria and gastrointestinal upset. The tremor occurs in about 20% of patients and can be managed by reducing the dose and/or administration of propranolol. Polyuria is alleviated by reducing the dose or adding amiloride. However, in the case of lithium-induced nephrogenic diabetes insipidus, lithium discontinuation may be necessary. Gastrointestinal side effects, such as nausea and diarrhea, occur in 10–20% of patients during the initial period of lithium use and usually disappear with further treatment (Ferensztajn-Rochowiak and Rybakowski 2023).

Among adversities appearing in the further period of lithium administration, the effects of lithium on weight gain, thyroid and parathyroid function could be mentioned. However, the most important could be those on the kidney function. Recently, it was demonstrated that lithium-induced weight gain, which was previously regarded as an important issue of long-term lithium therapy, does not make a significant problem (Gomes-da-Costa et al. 2022; Greil et al. 2023) The prevalence of lithium-induced goiter is estimated at 30–59%, based on over thirty years of observational studies. The risk of hypothyroidism is about 20%, mainly in the first years of lithium therapy and in subjects with a family history of thyroid dysfunction. We demonstrated that such a chance is similar to bipolar patients not taking lithium, which may show that bipolar illness can predispose them to hypothyroidism. In a study of BD patients receiving lithium for 3–47 years, higher concentrations of TSH and higher thyroid volume were found compared to BD patients not receiving lithium. However, the structural changes were unrelated to the hormones' concentrations (Kraszewska et al. 2019a). No connection between lithium treatment and antithyroid antibodies was found (Kraszewska et al. 2019b). In the group of 66 patients receiving lithium for 10–44 years, we did not observe differences in thyroid function between those taking lithium for less or more than twenty years (Kraszewska et al. 2015). The presence of hypothyroidism and/or large goiter during lithium therapy should not be the cause of lithium discontinuation but is the indication for thyroxine treatment, the dose could be consulted with an endocrinologist (Haissaguerre and Vantyghem 2022). Lithium therapy can be also associated with increased blood calcium and parathormone (PTH) levels (McKnight et al. 2012). In our study, the prevalence of hypercalcemia in 90 patients receiving lithium for 5–41 years (mean 16 years) was 10%, and the increase in PTH was found in three patients (Abramowicz et al. 2014). Therefore, the recommended procedure is to measure calcium and PTH levels once a year during long-term lithium treatment. In the case of primary lithium-induced hyper-parathyroidism, with the occurrence of clinical symptoms, the standard treatment as in primary hyperthyroidism could be implemented without the necessity of lithium discontinuation (Mifsud et al. 2020).

As to kidney function, there is an increasing risk of nephropathy in the course of interstitial nephritis after 10–15 years of lithium administration, reflected by a higher creatinine concentration and lower glomerular filtration rate (GFR). The risk factors for such renal impairment include high lithium concentrations, long duration of lithium treatment, older age, concomitant co-morbidities, lithium introduction after the age of 40, and initially lower GFR. The study from Poznan center comprising 80 patients aged 60 ± 11 years and receiving lithium for 5–39 (average 16) years showed decreased (< 60 ml/min/1.73 m^2^) GFR results in 38% of males and 16% of females (Rybakowski et al. 2012). In the International Group for the Study of Lithium-treated Patients (IGSLI) project, the lithium effect was evaluated in 312 patients coming from twelve collaborating centers, receiving lithium for 8–48 years (total 6142 person-years). Lithium treatment caused a gradual decrease in renal functioning by about 30% more than due to aging alone. There was a GFR decline of 0.7% per year of age and 0.9% per year of lithium treatment, both 19% more among female than male patients. One-third of the group had GFR < 60 ml/min/1.73 m^2^ at least once, mainly in those treated with lithium for more than 15 years and aged over 55. However, end-stage renal failure was not noticed (Tondo et al. 2017).

One of the recent population-based cohort studies showed no differences in mean annual decline in GFR between patients on lithium maintenance treatment and patients treated with other mood stabilizers (Clos et al. 2015). The second demonstrated lithium association with an increased risk of kidney impairment compared to valproate in the population of older adults (mean age 71 years), and the effect was more evident with higher lithium concentrations (> 0.7 mmol/l) (Rej et al. 2020). The prevalence of end-stage renal failure in lithium patients has been estimated at 0.015% (Aiff et al. 2014). There are also case reports describing reintroducing lithium treatment in BD patients after renal transplantation due to end-stage renal disease (Moss et al. 2014; Beasley et al. 2018).

In a five-year observation of four excellent lithium responders (three male and one female) receiving lithium for 22–45 years and having GFR < 50 ml/min/1.73 m^2^, we noticed that in three patients having GFR 47–48 ml/min/1.73 m^2^, the renal parameters did not show significant changes, and the patients continued lithium treatment as previously. The male patient with the lowest GFR (32 ml/min/1.73 m2) had a 14% decrease in GFR and a 10% increase in serum creatinine during this time. In him, the dose of lithium was decreased by one-third, and he was referred to a strict nephrological observation (Abramowicz et al. 2017).

The recommendations for monitoring renal status in patients treated longitudinally with lithium include the measurements of serum lithium, urea, electrolytes, and creatinine every 3 to 6 months. The patients should be referred to a nephrologist if GFR is < 30 ml/min/1.73 m^2^, and a progressive decline in GFR is observed (Davis et al. 2018). It should be avoided to combine longitudinally lithium with drugs that can increase nephrotoxic potential, e.g., nonsteroidal anti-inflammatory drugs, angiotensin-converting enzyme inhibitors, angiotensin receptor blockers, some antibiotics, and cytostatic and immunosuppressive drugs. Patients should not be allowed to have acute episodes of lithium intoxication. In the case of exacerbation of chronic kidney disease, the lithium dose should be reduced. A consideration of stopping lithium should be taken with great caution also because of the possibly associated suicide risk in patients with such pre-existing vulnerability. The overhasty discontinuation of lithium, sometimes recommended by some physicians, should be detrimental, since in most patients, any other mood stabilizer cannot effectively replace lithium. The continuation of lithium even in small doses is particularly important in excellent lithium responders in whom, after the drug discontinnuation, much of the benefit could be lost.

The recent recommendations for the prevention and management of lithium-induced kidney injury were described by Gitlin and Bauer (2023) and summarized in Table 2.

**Table 2:** Prevention and management of kidney injury during lithium treatment

Prevention	Management
Baseline assessment of renal function Using lithium in lowest effective dose In the first five years, lithium administration in one daily dose Avoiding high lithium levels and lithium intoxication Monitoring polyuria, in severe cases adding amiloride or hydrochlorothiazide	Monitoring of creatinine and GFR In case of GFR below 60 min/min or decline 5 ml/min/year or 10 ml/min/5 year: Monitoring 2–4 times/year Consider reducing lithium dose Referring to a nephrologist—explore other possible etiologies of renal insufficiency Lithium discontinuation and replacing with other mood stabilizer—very gradually should be a decision of last resort

## Beneficial effects of lithium administration

Treatment with lithium can bring additional fringe benefits that are not related to the prevention of mood episodes. The anti-suicidal effect of lithium therapy has been regarded as the most beneficial phenomenon of such treatment. It was amply evidenced in the 1990s by the studies of IGSLI investigators (Müller-Oerlinghausen et al. 1991, 1994, 1996) and confirmed in the meta-analyses of the twenty-first century (Baldessarini et al. 2006; Cipriani et al. 2013). Lithium’s anti-suicidal effect is not correlated with mood stabilization, which points to a specific aspect of lithium activity Lewitzka et al. (2015). Therefore, this effect, observed mainly after 2 years of lithium prophylaxis, can make an essential asset for patients being on lithium therapy. The suicide preventive effect of lithium extends not only to bipolar patients but also to those with unipolar depression (Cipriani et al. 2013).

Experimental evidence for the antiviral effect of lithium was provided more than forty years ago when the inhibition by lithium of the herpes simplex virus (HSV) replication was shown (Skinner et al. 1980). In a collaborative study of the Department of Adult Psychiatry, Poznan University of Medical Sciences, and the Department of Psychiatry of the University of Pennsylvania we conducted clinical research on labial herpes caused by HSV-1 in patients receiving lithium for prophylactic purposes. During lithium therapy in Polish BD patients with recurrent herpes, the completed cessation of recurrences occurred in 46% of subjects, and the general decrease in recurrence frequency was 64%. A better effect was observed in patients whose serum lithium concentration was higher than 0.65 mmol/l and whose intracellular (erythrocyte) lithium concentration exceeded 0.35 mmol/l. In American BD patients with labial herpes receiving lithium, the frequency of recurrences during 5 year treatment decreased by 73% compared to the 5 year pre-lithium period (Rybakowski and Amsterdam, 1991).

As herpes viruses are DNA viruses, it would also be interesting to know whether lithium may act on RNA viruses, including coronaviruses. Amsterdam et al. (1999), in a retrospective study including 236 patients with mood disorders, among those 177 taking lithium carbonate and 59 receiving antidepressants on a chronic basis, showed a statistically significant reduction in mean yearly rates of flu-like infections caused mainly through RNA viruses in lithium- but not antidepressant-treated patients. Shortly after the outbreak of the COVID-19 pandemic, Nowak and Walkowiak (2020) presented experimental data on the possible antiviral effect of lithium in coronavirus infections. Also, Murru et al. (2020), describing the antiviral effect of lithium, suggested its potential usefulness in patients with COVID-19 disease. However, the observations on the relationship between lithium and COVID-19 are not convincing. The most interesting is the research evaluating the relationship between the presence of COVID-19 infection and lithium levels in serum in 26,554 patients. It showed that the incidence of disease was significantly lower in those with lithium levels maintained within therapeutic limits (0.5–1.0 mmol/l) compared with those with lithium levels < 0.5 mmol/l. It is possible to see a parallel here to the relationship between the concentration of lithium and the intensity of its effect on the herpes virus (Rybakowski and Amsterdam, 1991). In patients with a therapeutic level of lithium, the incidence of infection was significantly lower compared with those using valproate (De Picker et al. 2022).

There can be an association between lithium treatment and a reduction of dementia risk, as was suggested by the results of population studies (Donix and Bauer 2016). The analysis of the Danish nationwide register of lithium prescriptions revealed that in patients with continued lithium treatment, the rate of dementia decreased to the same level as in the general population. This was not the case for persons treated with anticonvulsant drugs (Kessing et al. 2008). In another study, it was found that the continued treatment with lithium was also associated with a reduced rate of dementia in patients with BD, in contrast to such treatment with anticonvulsants, antidepressants, and antipsychotics (Kessing et al. 2010).

## Ultra-long-term lithium administration

In 2016, we described five patients (two men and three women, aged 64–79 years) with a good response to lithium treatment performed for 40–45 years. Their mean lithium concentration during the medication was 0.60–0.65 mmol/l (four subjects) and 0.7–0.8 mmol/l (one male). Both male patients presented signs of kidney impairment, but no worsening was observed in the last five years of observation. One woman with Hashimoto’s disease required treatment with levothyroxine. All patients had normal serum calcium concentrations. Also, their cognitive functions and professional activity were similar to age- and education-matched healthy persons. All patients played their family and social roles profitably. The initiation of lithium took place in the early stage of the illness, which could be an advantageous factor for the good prophylactic effect of lithium (Permoda-Osip et al. 2016).

In 2021, we described a successful 50 year lithium treatment case in a 79 year-old woman, which can be considered a model example of an excellent lithium responder (Ferensztajn-Rochowiak et al. 2021). Her prophylactic therapy with lithium began in 1970, at the age of 29, after her third depressive episode. Since then, she has maintained clinical remission, which enabled her to live a stable and satisfying life. The patient was professionally active until 65 years as an ophthalmologist. The renal examination performed in 2012 revealed an asymptomatic stage 2 chronic kidney disease. In 2013, the thyroid function and serum calcium concentration were normal. During her long-term lithium therapy, an elimination of viral respiratory infections and a significant reduction in labial herpes infections were observed. In this patient, the possible factors for a beneficial response to lithium treatment include a hyperthymic temperament and an early start of lithium administration.

Below, we present a case series of the subsequent three patients who have reached their 50th anniversary of prophylactic lithium treatment in 2022 and 2023.

### Patient 1

The patient is a 73-year-old male born in 1949. The diagnosis of bipolar disorder was established in 1971, at the age of 22, with the first presentation of a manic episode. Before lithium treatment, the patient was hospitalized several times due to manic and mixed episodes. Since February 1972, he has continuously received lithium carbonate in the outpatient clinic until today, with a good response. Since the introduction of lithium in 1972, the doses were maintained between 1000 and 1500 mg/day. However, they were diminished to 500 mg/day in the last few years. For the whole treatment period, no manic episodes occurred, however, at least three depressive episodes appeared: the first one lasted for a brief period and resolved very quickly after amitriptyline 25 mg; the second required hospitalization in 1981; the third one was treated with mianserin 60 mg, later changed to dibenzepine, resolved after few months and then patient remained only on lithium monotherapy. Since the 1990s, mild to moderate depressive symptoms occurred periodically, and sometimes for several weeks or months, when an antidepressant had been added to the therapy (i.e., clomipramine, mianserin, paroxetine, venlafaxine). However, with higher dosages of antidepressants, the mental state revealed some features of mixed state (irritability, flying thoughts). Always after achieving euthymia, the patient returned to lithium monotherapy. Sporadically, the transient symptoms of agoraphobia (e.g., a fear of being in a crowd or going to a church) appeared.

The patient had a secondary education and worked in a furniture factory until retirement. He has been married for over 50 years, has three children and three grandchildren, and has remained in a good, supportive relationship with his wife and family until now. The family history shows that his sister was treated for dysthymia, his niece was treated for depression, and his cousin was diagnosed with bipolar disorder. The somatic co-morbidity included migraines since the patient’s 20 s and hypertension after his 60 s, treated with ramipril, 5 mg.

The serum lithium concentration has been between 0.60 and 0.65 mmol/l. The main side effects in the first years of treatment were moderate sexual dysfunctions and hand tremors (with higher doses of lithium). The renal status corresponds to stage 3 chronic kidney disease. However, no signs of the progression of renal damage were noticed in the last ten years. It is noteworthy that the patient has a congenital renal malformation in the form of duplex kidney. The levels of thyroid hormones and anti-thyroid antibodies have remained normal throughout treatment. However, a thyroid ultrasound examination in 2013 revealed thyroid enlargement, four solid nodules, and normal vascular flow, which resulted in recommendations for regular follow-ups at the endocrinology outpatient clinic without any pharmacological or surgical intervention.

### Patient 2

The 73-year-old female born in 1950 has been treated with lithium since May 1973, when she was 23 years old. The lithium was started during her hospitalization caused by a manic episode that occurred after the birth of her first child. In 1976 and 1985, she had two breaks in taking lithium during the second and third pregnancy. Within a week after her second delivery, she developed manic symptoms, which resulted in hospitalization, however, lithium reintroduction resulted in remission. During the lifetime course of illness, the patient, for the most time, remained stable and euthymic. However, several manic episodes without psychotic features occurred, usually after essential events in life (only one required hospitalization), and resolved within a few weeks after combined treatment with haloperidol. Since 2000, perphenazine, and then in 2013, risperidone, were added to lithium treatment due to recurrent manic symptoms. Nevertheless, she was professionally active throughout her life and worked as a biologist and, from 1991, as an English teacher. She has remained in a stable and supportive marriage, ran a home for many years, raised three children, and lived a satisfying life.

From the beginning, the patient tolerated lithium treatment very well, and no side effects occurred. The lithium doses within the first 20 years of treatment were 1000 mg/day, then reduced to 750 mg/day, due to increasing serum lithium levels during remission, amounting to 0.9 mmol/l. However, the average lithium concentration during the whole period of lithium treatment was maintained within the 0.6–0.7 mmol/l range.

The patient has remained under the care of a rheumatologist since the 1980s due to ankylosing spondylitis. After 40 years of lithium treatment, stage 2 chronic kidney disease was diagnosed, and the patient was periodically seen by a nephrologist. The thyroid function remains normal, showing no abnormalities.

### Patient 3

The 88-year-old male, born in 1935, has been treated with lithium since November 1973. He had three hospitalizations, the last one in 1975. He has been in remission for 5 years since 1975. In 1980, he decided to discontinue the drug, which resulted in a manic episode. After hospitalization and reintroduction of lithium treatment, the remission was achieved again. Since that time, he remained under regular care of the outpatient clinic, taking lithium systematically and maintaining a state of remission until now. He has been living a stable family life and has a wife, children and grandchildren. Also, he was professionally active as a teacher until retirement.

Between 1975 and 1993, he received 1250 mg/day of lithium carbonate, then reduced to 1000 mg/day and eventually to 750 mg/day in 2003. Serum lithium levels were maintained between 0.5 and 0.8 mmol/l. While taking a dose of 1250 mg of lithium, symptoms such as trembling hands and increased thirst periodically occurred.

In 1983, he underwent a heart attack and, in 2011, a transient ischemic attack without any remaining neurological complications. The last renal assessment revealed stage 2/3 chronic kidney disease at a stable level for the past ten years. Thyroid function remained normal throughout the treatment.

## Conclusions

Several elements could be identified in summarizing the characteristics of our ultra-long-term lithium-treated patients described above. The first episode in the course of disease was manic in all three of them. The diagnosis was bipolar I disorder, and the clinical course of illness before starting lithium treatment was characterized as episodic with periods of precise intervals of remission. Lithium treatment was started in the first years of the disease. Therefore, there was a short duration of illness before the drug introduction. No rapid-cycling features, the absence of psychotic symptoms, and no evident psychiatric comorbidity were observed during the lifetime of all patients. The patients reported no childhood trauma or traumatic life events during childhood, and all had stable and adequate social support. Two patients had hyperthymic personalities and all showed good compliance. However, a positive family history of bipolar disorder could be identified only in one patient. The mean serum lithium concentration was in the range of 0.5–0.8 mmol/l, according to recent guidelines (Nolen et al. 2019), which could partly lessen the possible renal and cognitive problems.

The patients' life history can be characterized by a stable lifeline, i.e., long-term marriage, no divorce, having healthy children, and professional activity until retirement, which could relate to Paul Grof’s definition of excellent lithium responders. During lithium treatment, no suicidal attempt in any of the patients was observed. Despite an increased risk of developing dementia in bipolar disorder (Diniz et al. 2017), all patients were intellectually competent. This finding can relate to our study showing that patients described as ELR had normal levels of cognitive functions (Rybakowski and Suwalska 2010). In addition, even though the mean of 20 years duration of the illness, they have serum BDNF similar to healthy subjects, despite claims that a decline in BDNF can make a marker for the late stage of the illness (Kauer-Sant’ Anna et al. 2009).

Given the mean age of our patients was 78 years, it could be underlined that life expectancy in bipolar disorder is significantly reduced, reaching average of 66 years (Chan et al. 2022). Worth mentioning is also a paucity of chronic and uncompensated states of somatic co-morbidity in our patients, which is widely described in bipolar disorder, including cardiovascular, metabolic, neuroendocrine, and inflammatory diseases (Seeman et al. 2001).

It should be emphasized that the cases described represent a small selection of patients with a very positive course—in our experience also due to the early use of lithium. Nevertheless, it must be said that many other factors also contributed to this positive course (e.g. the good, stable social conditions). Therefore, for clinical care, lithium should be considered and used at an early stage and prescribed on a long-term basis once the response has been determined. For patients who do not respond well, however, other pharmacological options may be recommended.

In conclusion, we would like to promote the usefulness, efficacy, and safety of ultra-long-term lithium therapy. Adequate patient selection should be kept in mind, pointing at subjects predisposed to a good response to lithium. We can recommend the early administration of lithium in the course of the disease. Appropriate monitoring should be carried out, taking into account regular measurements of lithium concentrations, electrolytes, calcium, kidney, and thyroid function. In case of any concern, a suitable intervention should be given.

Last but not least, despite the therapeutic and prophylactic control of mood episodes, lithium treatment can bring additional beneficial effects. The most important is the prevention of suicides and probably dementia. Some patients can also take advantage of the antiviral properties of lithium.

## Data Availability

Not applicable.
