# Correction: Effectiveness of ultra-long-term lithium treatment: relevant factors and case series

**DOI:** 10.1186/s40345-024-00359-2

**Published:** 2024-10-26

**Authors:** Ewa Ferensztajn-Rochowiak, Ute Lewitzka, Maria Chłopocka‑Woźniak, Janusz K. Rybakowski

**Affiliations:** 1 Department of Adult Psychiatry, Poznan University of Medical Sciences, Poznan, Poland; 2 Department of Psychiatry and Psychotherapy, Universitätsklinikum Carl Gustav Carus, Dresden, Germany; 3 TUD Dresden University of Technology, Dresden, Germany

**Correction to: International Journal of Bipolar Disorders (2024) 12:7**

10.1186/s40345-024-00328-9

In this article (Ferensztajn-Rochowiak et al. 2024), the affiliation ‘TUD Dresden University of Technology, Dresden, Germany’ for the author Ute Lewitzka was missing. The correct affiliation for the author Ute Lewitzka is given below.

Ute Lewitzka^2,3^

^2^Department of Psychiatry and Psychotherapy, Universitätsklinikum Carl Gustav Carus, Dresden, Germany.

^3^TUD Dresden University of Technology, Dresden, Germany.
